# Extensive transcriptome data providing great efficacy in genetic research and adaptive gene discovery: a case study of *Elymus sibiricus* L. (Poaceae, Triticeae)

**DOI:** 10.3389/fpls.2024.1457980

**Published:** 2024-09-19

**Authors:** Yanli Xiong, Daxu Li, Tianqi Liu, Yi Xiong, Qingqing Yu, Xiong Lei, Junming Zhao, Lijun Yan, Xiao Ma

**Affiliations:** ^1^ College of Grassland Science and Technology, Sichuan Agricultural University, Chengdu, Sichuan, China; ^2^ Sichuan Academy of Grassland Sciences, Chengdu, Sichuan, China

**Keywords:** adaptability, transcriptome, genome skimming, SNPs, WGCNA, *Elymus sibiricus*

## Abstract

Genetic markers play a central role in understanding genetic diversity, speciation, evolutionary processes, and how species respond to environmental stresses. However, conventional molecular markers are less effective when studying polyploid species with large genomes. In this study, we compared gene expression levels in 101 accessions of *Elymus sibiricus*, a widely distributed allotetraploid forage species across the Eurasian continent. A total of 20,273 high quality transcriptomic SNPs were identified. In addition, 72,344 evolutionary information loci of these accessions of *E. sibiricus* were identified using genome skimming data in conjunction with the assembled composite genome. The population structure results suggest that transcriptome SNPs were more effective than SNPs derived from genome skimming data in revealing the population structure of *E. sibiricus* from different locations, and also outperformed gene expression levels. Compared with transcriptome SNPs, the investigation of population-specifically-expressed genes (PSEGs) using expression levels revealed a larger number of locally adapted genes mainly involved in the ion response process in the Sichuan, Inner Mongolia, and Xizang geographical groups. Furthermore, we performed the weighted gene co-expression network analysis (WGCNA) and successfully identified potential regulators of PSEGs. Therefore, for species lacking genomic information, the use of transcriptome SNPs is an efficient approach to perform population structure analysis. In addition, analyzing genes under selection through nucleotide diversity and genetic differentiation index analysis based on transcriptome SNPs, and exploring PSEG through expression levels is an effective method for analyzing locally adaptive genes.

## Introduction

Local adaptation occurs when divergent selection outweighs random genetic drift and the equalizing effect of gene flow between populations (Kawecki and Ebert, 2004). These premises suggest that certain widely distributed wild plants show distinctive local adaptations caused by restricted gene flow between populations due to geographical and environmental isolation. Multiple selection factors interact with geographic distance between populations to shape the evolutionary dynamics of species by restricting gene flow (López-Goldar and Agrawal, 2021). In addition to the molecular perspective and adaptive effects, a comprehensive study of local adaptation in species should also consider ecological factors and the co-evolution of plants and their environment. For example, local adaptation along natural gradients such as latitude and altitude, which have been extensively studied, may be the result of the combined influences of different ecological effects on selection and gene flow (López-Goldar and Agrawal, 2021; Tiffin and Ross-Ibarra, 2014).

Compared to neutral regions, where selection is not expected, regions of genes and linked loci under divergent selection are expected to experience stronger selection pressures (Limborg et al., 2012). The adaptive significance of existing polymorphic sites is often difficult to estimate, as they may not be the direct targets of selection, but rather by-products of hitchhiking effects (Zhang et al., 2024). Large-scale transcriptome sequencing can help us study functional genetic variation, thereby increasing the chances of detecting natural selection, as functional genetic variation is expected to be more directly influenced by natural selection and result in a population-specific expression (Tang et al., 2021). The regulation of gene expression is a complex and intricate process, and studies of the evolution of gene expression have shown significant variability in the variability of regulatory elements both within and between natural populations (Shi et al., 2012). However, studying adaptive evolution at the level of gene expression is only relevant for specific genes or sets of co-expression gene. Given that the environment always influences gene expression, this implies that general evolutionary patterns of gene expression are not necessarily adaptive (Signor and Nuzhdin, 2018). Common garden experiments test how heritable traits are influenced by natural selection (leading to natural variation) and ultimately affect optimal outcomes for population persistence (Schwinning et al., 2022). The natural variation observed could be attributed to the prolonged acclimatization processes that occur in response to the particular environmental conditions of the sites where the species occur.

*Elymus sibiricus*, the model species of the genus *Elymus*, is widely distributed across the Eurasian continent and exhibits significant genetic diversity and phenotypic variance (Xiong et al., 2021). The extensive habitat diversity has contributed to the unique adaptability of *E. sibiricus* ecotypes across different geographic groups (Han et al., 2022). Due to its remarkable adaptability to the high altitude environment, superior nutritional value and ease of cultivation, *E. sibiricus* is the dominant forage crop and is extensively cultivated in the artificial forage grasslands of the Qinghai-Tibetan Plateau. Nevertheless, the unavailability of the reference genome hampers genetic research on *E. sibiricus*, resulting in previous studies relying solely on molecular markers such as genomic SSRs (Xiong et al., 2021; Lei et al., 2014), EST-SSRs (Zhang et al., 2019; Zheng et al., 2020), and chloroplast/mitochondrial SSRs (Lei et al., 2014; Xiong et al., 2022), to investigate the population structure and adaptive evolution of the species. Due to their limited coverage, these markers can only reveal specific genetic variations. Considering their larger number and wider distribution, SNP markers are more suitable for population genetic research (Sun et al., 2020). Some SNPs derived from simplified genome sequencing have been used to study the population structure of *E. sibiricus* (Han et al., 2022). However, a significant proportion of these markers are located in non-functional regions. Furthermore, mapping SNPs located in genic regions to specific genes is challenging due to the lack of comprehensive reference genomic information. In this context, the use of transcriptome data allows a thorough investigation of functional regions and genes associated with adaptive traits, and the detection of sequence variation and expression levels within these regions. SNP-based studies of the transcriptome can provide a wealth of SNP markers, and these variants can affect the coding sequence of proteins, thus affecting gene expression and function. In addition, the abundance of SNP markers in the transcriptome allows accurate assessment of genetic variation and population structure (Yang, 1998). However, issues of widespread purifying selection in the transcriptome and concerns about sample size point to the need for comparisons between mRNA and established DNA-based methods (Thorstensen et al., 2020; Pratlong et al., 2015; Thorstensen et al., 2021). Genome skimming sequencing is a cost-effective protocol for obtaining genome-level variation and has shown excellent potential for phylogenetics, species identification, and genetic diversity assessment (Richter et al., 2015; Trevisan et al., 2019). Furthermore, SISRS software allows the identification of phylogenetically informative sites across the genome by skimming sequencing data without reference genome information (Schwartz et al., 2015; Literman et al., 2023), allowing us to perform population variation calling at the DNA level.

This study included 101 wild *E. sibiricus* accessions from both domestic and international sources, covering the primary distribution regions of *E. sibiricus*. The transcriptomes of these collected accessions were sequenced, and the full-length transcriptome of *E. sibiricus* was assembled using previous research data to construct a reference sequence. The aims of this study included: (1) studying population genetic analysis using transcriptome SNPs and expression levels; (2) analyzing population (geo-group)-specific expression genes (PSEG), investigating genes related to adaptability of *E. sibiricus*; (3) using genome skimming data from 101 accessions as reported by Xiong et al. (2023) to identify homologous sequences at the genome level (Schwartz et al., 2015) and compare their effectiveness in population structure studies with transcriptome data (both transcriptome SNPs and expression levels). This study provides a comprehensive analysis of the population structure of *E. sibiricus*, including transcriptome SNPs, expression levels and genome-level homologous sequences, which could provide a genetic research mode in polyploid species without genomic information. It will also identify the functional genes that contribute to the differentiation of geographic groups of *E. sibiricus*.

## Results

### Full-length transcriptome assembly, SNPs calling from the transcriptome and genome skimming data

The reference transcriptome of *E. sibiricus* was assembled, comprising 6982 contigs with an N50 of 42,573 bp and a GC content of 48.08% (**Table 1**). Using this reference transcriptome, 20,273 high quality SNPs were identified from 101 *E. sibiricus* accessions. Furthermore, the genome skimming data were used to assemble the *E. sibiricus* composite genome, which consisted of 33,234 contigs with a total length of more than 3.8 million bp, covering more than 0.05% of the 6.8 Gb nuclear genome. Among these contigs, 1525 had a length of more than 200 bp. A total of 72,344 SNPs were identified based on genome skimming data from 101 *E. sibiricus* accessions.

**Table 1:** The quality assessment of the assembled reference transcriptome of *E. sibiricus*.

Number of contig	Largest contig	contigs (>= 50 kbp)	N50	L50	N’s per 100 kbp	GC content
6982	170,783	342	42,573	448	0	48.08%

### SNPs derived from the transcriptome data exhibited higher discriminatory ability for various geo-groups of *E. sibiricus*

**Figure 1:**
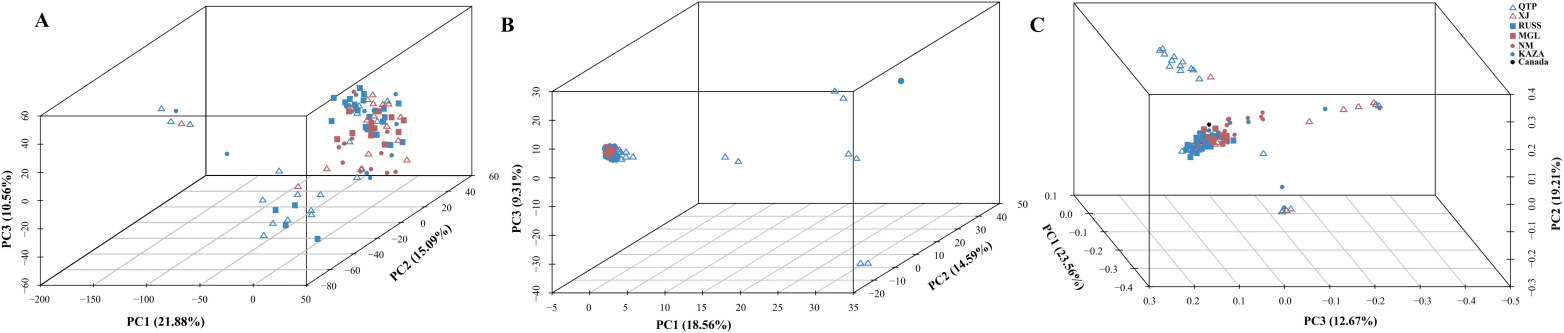
The PCA plots based on the gene expression levels **(A)**, SNPs called from the genome skimming [SNPs-GS, **(B)**] data, and transcriptome SNPs **(C)**. QTP, Qinghai-Tibet Plateau, China; KAZA, Kazakhstan; MGL, Mongolia; NM, Inner Mongolia, China; RUSS, Russian; XJ, Xinjiang, China.

**Figure 2:**
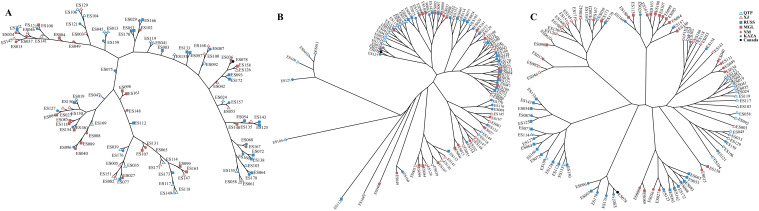
The hierarchical clustering based on Euclidean distance using the expression levels **(A)**; Maximum likelihood tree using SNPs-GS **(B)** and transcriptome SNPs **(C)** of 101 *E. sibiricus* accessions.

The PCA plots and phylogenetic trees based on three data sets (transcriptome SNPs, the expression levels, and SNPs-GS) were combined to reveal the population structure of the tested *E. sibiricus* accessions (**Figures 1**, **2**). In general, a greater discriminatory power of SNP data (both transcriptome SNPs and SNPs-GS) to discriminate different geo-groups was observed in PCA plots. Specifically, the PCA plot constructed using SNPs-GS could distinguish several *E. sibiricus* accessions from the QTP (**Figure 1B**), while the PCA plot constructed using transcriptome SNPs could distinguish more *E. sibiricus* accessions from the QTP and other regions (**Figure 1C**). However, based on expression levels, the tested *E. sibiricus* accessions had a mixed population structure (**Figure 1A**). In addition, the phylogenetic analysis also showed a greater discriminatory ability of transcriptome SNPs for distinguishing *E. sibiricus* accessions from different geo-groups (**Figure 2**), indicating the suitability of SNP data from transcriptome sequencing for further population structure analysis compared to expression levels or SNPs-GS.

### Genetic diversity indices using transcriptome SNPs

As transcriptomic SNPs were more effective in revealing the population structure of *E. sibiricus* (**Figures 1**, **2**), we subsequently used transcriptomic SNPs to perform further genetic studies. In summary, XJ and SC/XZ had the highest (0.3248) and lowest (0.3186) MAF values (**Table 2**), respectively. For O_het, except for the QH geo-group, all groups had values greater than 0.6, with the MGL geo-group having the highest value (0.625). However, the QH geo-group had the highest value of E_het (0.3894). The O_het values of all the geo-groups were greater than the E_het values.

**Table 2:** Genetic diversity indices of each geo-group of *E. sibiricus*.

Geo-groups	MAF	O_het	E_het
Canada	0.3096	0.6191	0.3096
SC	0.3186	0.6188	0.3651
XJ	0.3248	0.6124	0.3824
RUSS	0.3237	0.6189	0.3786
QH	0.3218	0.5881	0.3894
NM	0.3240	0.6207	0.3758
MGL	0.3258	0.6250	0.3779
KAZA	0.3238	0.6012	0.3865
XZ	0.3186	0.6176	0.3605

MAF, minor allele frequency; O_het, observed heterozygosity; E_het, expected heterozygosity.

### Gene flow and differentiation among geo-groups based on transcriptome SNPs

**Figure 3:**
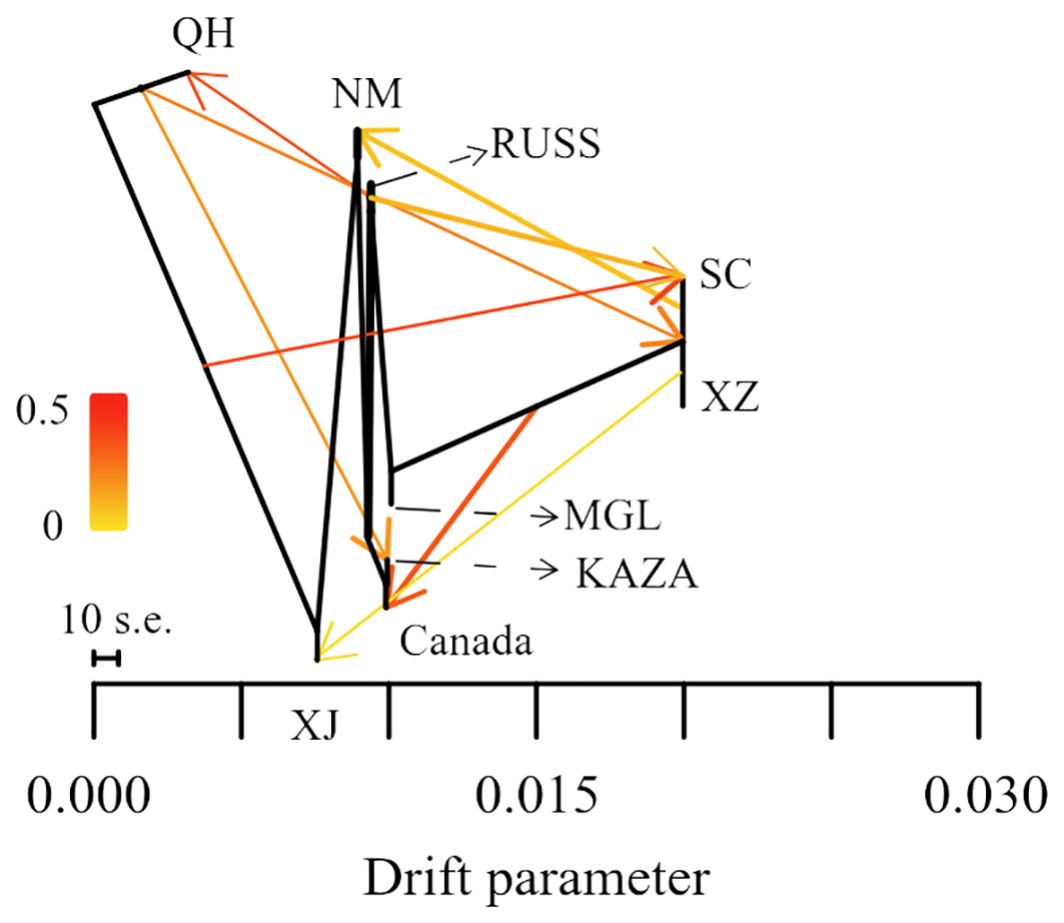
The gene flow among nine geo-groups of *E. sibiricus*.

The result of the gene flow analysis showed that the optimal K value (migration edges) was eight, indicating the presence of eight gene flow events between the nine geo-groups of *E. sibiricus* (**Supplementary Figure S1**). This was consistent with the generally low Fst values between these geo-groups (**Supplementary Figure S2**). The SC geo-group had the highest number of gene flow events, including gene flows from QH and RUSS to SC, and SC to NM in the early stages (**Figure 3**). Only a single gene flow event was detected for the Canada, NM, KAZA, and XJ geo-groups. In contrast, no gene flow was observed between MGL and the remaining geo-groups.

**Figure 4:**
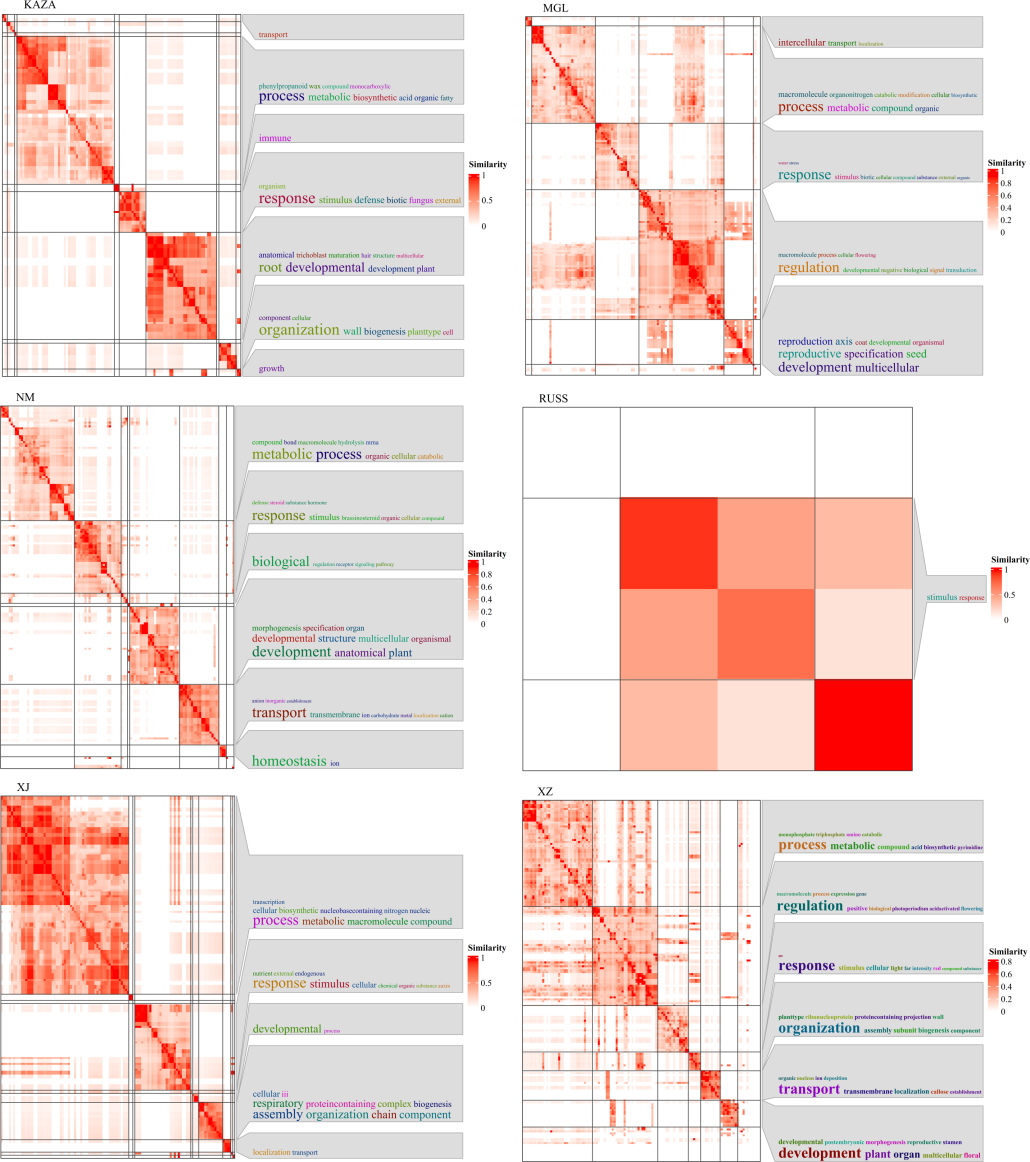
The GO annotation result of genes under selection in each geo-group of *E. sibiricus*.

We further integrated the Pi values for each geo-group and the pairwise Fst values to identify the selected regions and associated genes. As the Canada geo-group contained only a single accession, it was excluded from this analysis. A total of 12, 267, 3, 81, 1, 54, 824, and 259 genes were identified in the XZ, XJ, SC, RUSS, QH, NM, MGL, and KAZA geo-groups, respectively. Three and one genes selected in the SC and QH geo-groups, respectively, could not be annotated in the GO database. The selected genes from all six geo-groups were involved in the “development” and “metabolic” processes (**Figure 4**). With the exception of the NM and KAZA geo-groups, the genes under selection in other geo-groups were also involved in the homeostasis process. In addition, the genes under selection in each geo-group showed responsiveness to specific stresses or stimuli, potentially contributing to the cumulative variation experienced during the long-term adaptation process to environmental factors.

### Population differentiation from the perspective of gene expression

Using the expression levels of all genes cannot effectively resolve the population structure of *E. sibiricus* (**Figures 1A**, **2A**). Therefore, we attempted to analyze genes specifically expressed in each geo-group to investigate population differentiation from the standpoint of gene expression. A total of 414, 1899, 1797, 498, 1529, 3295, 556, and 4499 genes were specifically expressed in KAZA, MGL, NM, QH, RUSS, SC, XJ, and XZ geo-groups, respectively. The heatmap results indicated that the specifically expressed genes in each geo-group showed a significant difference in expression profiles (**Supplementary Figure S3**).

We also performed GSVA (gene set variation analysis) to identify differentially enriched gene sets in each geo-group compared to the others. Several GO terms related to plant energy metabolism processes were identified, including GO:0052855 and GO:0047453, which are associated with NAD(P)H-hydrate dehydratase activity and were significantly up-regulated in the KAZA geo-group. In addition, GO:0033178 (related to ATPase complex) and GO:0006086, GO:0006637, and GO:0047617 (related to acyl-CoA metabolic process), were significantly upregulated in the MGL geo-group. Furthermore, several GO terms related to carbohydrate metabolism pathways were discovered, including GO:0009052, GO:0061615, and GO:0003872 in the MGL geo-group, GO:0019673, and GO:0008446 in the RUSS geo-group, GO:0006486, GO:0008378, and GO:0019255 in the SC geo-group, GO:0004576 in the XJ geo-group, GO:0019375 and GO:0047714 in the XZ geo-group. Although the plants were grown under controlled growth conditions, some GO terms related to stress tolerance were also detected for some geo-groups. For example, GO:0009408 and GO:0031990, which are associated with heat stress, were up-regulated in the KAZA geo-group, GO:0010225 (response to UV-C) and GO:0009411 (response to UV) were down-regulated in the MGL and SC geo-groups, respectively. GO:1901700 (response to oxygen-containing compound) and GO:0071215 (cellular response to abscisic acid stimulus) were downregulated in the SC geo-group. In particular, six GO terms (GO:0006833, GO:0015793, GO:0015254, GO:0015250, GO:0009992, and GO:0016798) related to water transport were significantly downregulated in the XJ geo-group.

**Figure 5:**
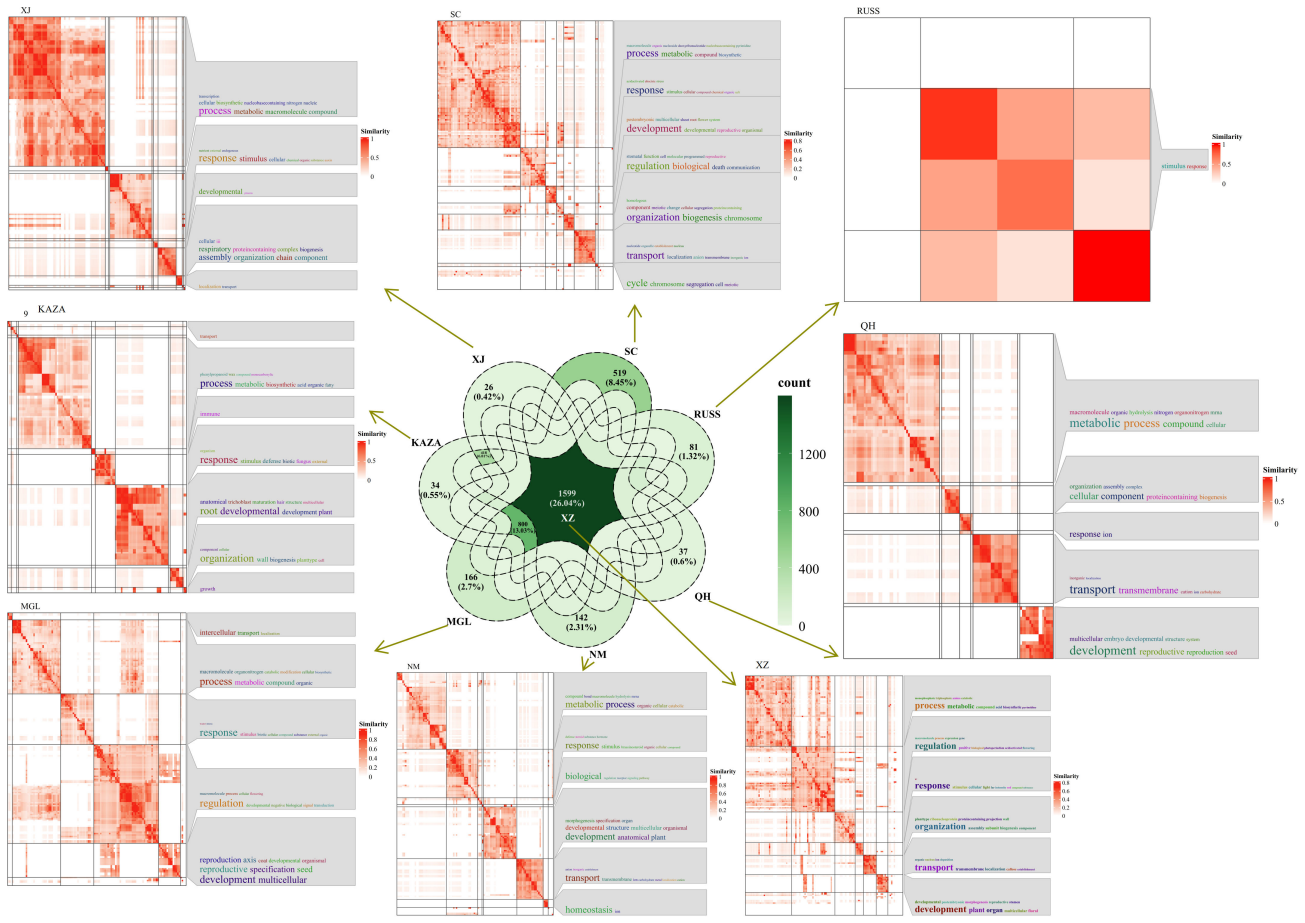
The Venn diagram of population-specifically-expressed genes between two geo-groups, and the GO annotation results of these shared specific-expressed-genes in each geo-group.

The PSEG were further analyzed between two geo-groups. The analysis revealed that the XZ geo-group had the highest number of shared specifically expressed genes shared with the other geo-group, with a total of 1599 genes (**Figure 5**). GO annotation results indicated that some of these genes were involved in the response process to UV (**Figure 5**). In addition, the common specifically expressed genes in the QH, NM and SC geo-groups were found to be responsive to ion, especially in the SC geo-group where the genes were responsive to salt and involved in ion transport process.

### WGCNA analysis can help identify potential regulators of PSEG

**Figure 6:**
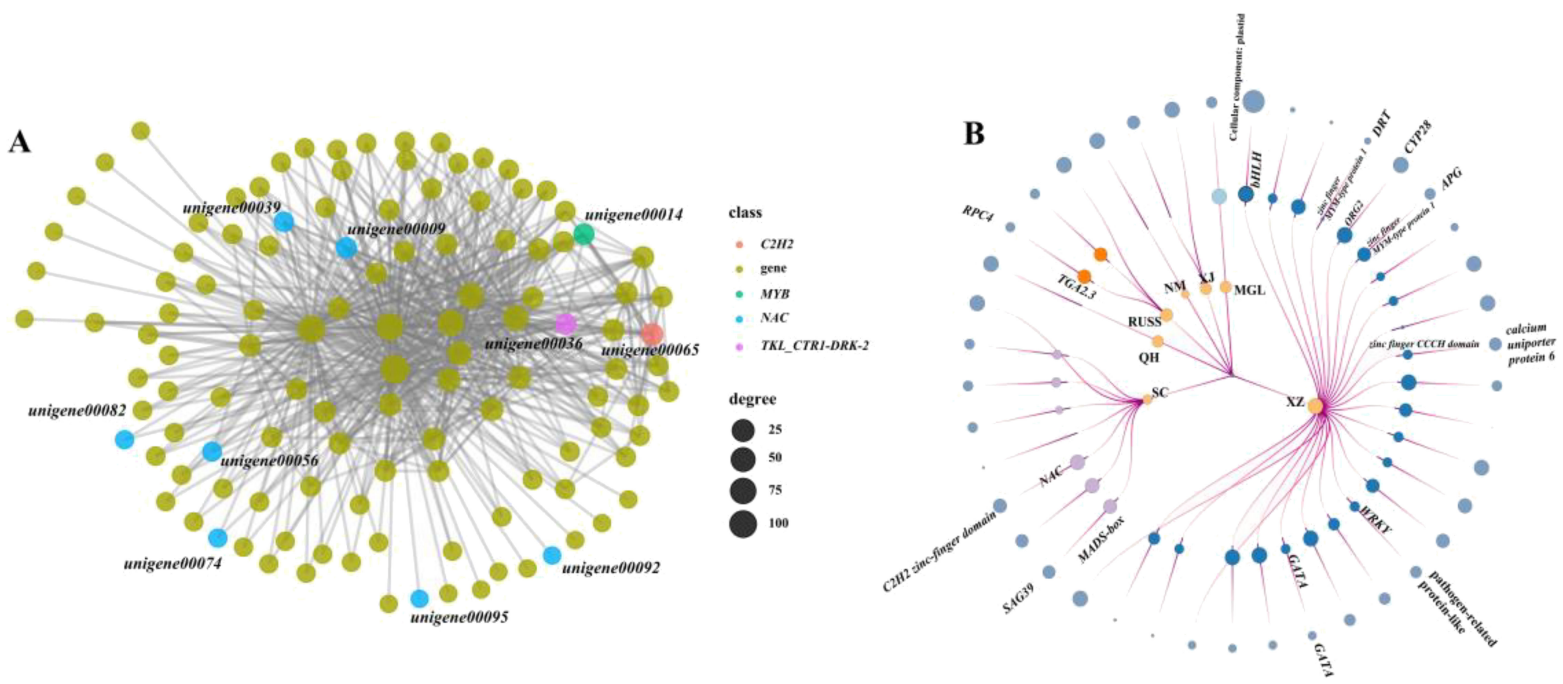
The node-link graph showing the regulating relationship between the population-specifically-expressed genes and the TFs. **(A)** regulatory network between TFs and genes in black model; **(B)** the node-link graph of PSEGs, the circles from the inside to out represent geo-groups, TFs and population-specific genes, respectively. The circle size indicates the number of times that the genes regulating or be regulated.

In this study, we performed a weighted gene co-expression network analysis (WGCNA) to identify the possible core regulatory genes in PSEG. The result showed that all the co-expressed genes could be assigned to 46 modules (**Supplementary Figure S4**). The regulatory networks in each model identified by WGCNA analysis were constructed and an extensive regulatory relationship between TFs such as NAC, MYB, C2H2, and downstream genes was found in the black model (**Figure 6A**). Furthermore, we also identified the potential TFs that have the ability to regulate the expression of PSEG in each geo-group (**Figure 6B**). It is important to note that the genes in the QH, XJ, and NM geo-groups do not function as regulatory genes. Conversely, in the SC geo-group, we discovered two TFs (*MADS-box* and *NAC*) that play a regulatory role in the genes encoding the senescence-associated protease (SAG39) and the C2H2-finger domain. In addition, we identified more regulatory TFs in the XZ geo-group, including two genes belonging to the zinc finger family. Notably, our analysis also revealed that some PSEGs are regulated by multiple TFs. For example, one gene, *DNA damage-repair/toleration* (*DRT*), specifically expressed in the XZ geo-group, was regulated by six TFs. These findings highlight the importance of using transcriptome data to effectively identify potential population-specific regulatory genes that may be associated with their own local adaptation.

## Discussion

To the best of our knowledge, this study represents the first attempt to assess the effectiveness of three methods - expression levels, transcriptome SNPs, and SNPs-GS - for analyzing population structure in a species lacking a reference genome, with a focus on *E. sibiricus*. The results showed that SNPs, especially transcriptome SNPs, were most effective in analyzing the population structure of *E. sibiricus*. This method identified numerous genes associated with local adaptation in *E. sibiricus*. In addition, although expression levels were less effective in population structure analysis, a significant number of PSEGs were also detected. Their annotations suggested their relevance to local environmental adaptation. Furthermore, by performing WGCNA analysis, some potential regulatory genes of the PSEGs were identified. Thus, we propose that in the absence of a reference genome, the use of transcriptome data (including both expression levels and SNP data) can reveal genes related to local adaptation and evolution of a species.

### Transcriptome SNPs is an effective strategy in population analysis

SNP markers have several advantages over other markers, including ease of development and deployment. However, using SNP markers to study the population structure of certain species, particularly polyploidy species in the absence of reference genome information, can be challenging. Most previous studies have relied on genome skimming methods to obtain plastid sequences and nuclear ribosomal genes (Trevisan et al., 2019; Liu et al., 2021b). However, these approaches have only revealed limited genetic variation, which is insufficient for a comprehensive understanding of the true population structure (Del Valle et al., 2020). It is possible to jointly *de novo* assemble and identify evolutionarily informative sites from genome skimming data, as demonstrated for species identification in buffalo fish (Literman et al., 2023) and phylogenomic reconstruction in Leishmania (Harkins et al., 2016). Notably, there are no reports of genome skimming data being used to identify evolutionarily informative sites within species. Given the significant variation among the wild *E. sibiricus* accessions used in our study, we attempted to use genome skimming data to detect evolutionarily informative sites and analyze the population structure of *E. sibiricus*. The assembled composite genome covered approximately 0.05% of the *E. sibiricus* nuclear genome (Xiong et al., 2021). Although the coverage of typical simplified genome sequencing is typically around 5% (Literman et al., 2023), it remains a challenge to effectively assemble and align genome sequencing data, as well as complicating the annotation of variant sites, in the absence of the reference genome for population structure analysis. In addition, the PCA analysis distinguished only a few *E. sibiricus* accessions from Qinghai that showed significant differences from other accessions (**Figure 1**), while the phylogenetic tree results show that the accessions from the Qinghai-Tibetan Plateau (in purple) are generally clustered together, while the accessions from the Xinjiang are mainly clustered in three branches in the lower right corner (**Figure 2**). This suggests that in the absence of genome information, using genome skimming data to identify evolutionarily informative sites for population structure analysis is a viable alternative. However, it is essential to adequately increase the sequencing depth and propose more efficient algorithms for the analysis of short-read sequencing data to accurately infer genetic structure, especially for polyploid species with large genome sizes.

Data from coding genes allow for quality control steps that help to distinguish biological patterns from technical artefacts, enabling SNP analysis data to be tested against neutral expectations at multiple levels, outperforming typical outlier approaches (De Wit et al., 2015). Many researchers (Wang et al., 2021; Mossion et al., 2022) have successfully developed transcriptome SNPs for the analysis of population structure and genetic diversity. Furthermore, the power of transcriptome SNPs to study intraspecific relationships in *Camellia sinensis* has been shown to exceed that of low-copy nuclear genes (Cheng et al., 2023). In this study, the higher effectiveness of transcriptome SNPs in deciphering the population structure of *E. sibiricus* from different locations compared to expression levels and SNPs-GS (**Figures 1**, **2**) was also found. On the other hand, transcriptomic SNPs may be concentrated in regions of active gene expression where variation and recombination may be more frequent, so the present study detected frequent gene flow (**Figure 3**) and generally small pairwise Fst coefficients between geo-groups using transcriptomic SNPs (**Supplementary Figure S4**). This is consistent with a previous research using chloroplast markers (Xiong et al., 2022). However, this seems to differ from the results of previous studies using whole chloroplast genomes (Xiong et al., 2023) and nuclear makers (Han et al., 2022): their results show that gene flow between geo-groups of *E. sibiricus* is relatively low due to the existence of geographic barriers. This may be because transcriptome SNPs capture more recent or small-scale gene flow than chloroplast or nuclear DNA, as these data provide more detailed genetic information (Chen et al., 2021; Tepolt and Palumbi, 2015).

### The combination of transcriptome SNPs and expression levels can help understanding adaptive mechanism

Transcriptome SNP can reduce complexity and provide more accurate functional annotation than reduced representation of genomic DNA libraries. This is considered an important method for identifying genes associated with local adaptation for population genomic analyses in non-model plants (Cheng et al., 2023; Liu et al., 2021a). In this study, the Pi and pairwise Fst values based on transcriptome SNPs were used to identify genes under selection within each geo-group. The majority of these genes are involved in the regulation of homeostasis, growth, and developmental processes (**Figure 4**). While some genes under selection in each geo-group are involved in various stress processes, only the NM geo-group had genes annotated as responding to ion stress processes. This is noteworthy because the NM geo-group is an area with severe saline alkali soils, and *E. sibiricus* from this region shows stronger salt tolerance (Chen et al., 2023). However, no adaptive genes related to local climatic or environmental characteristics (such as the arid climate of Xinjiang and the high altitude of Tibet) were detected in other geo-groups, possibly due to the frequent gene flow events between *E. sibiricus* geo-groups (**Figure 3**).

In addition to transcriptome SNPs, variation in gene expression levels is also an important source of adaptive traits, as gene transcription levels serve as the medium for gene coding information and final phenotypes (Alonso-Blanco et al., 2009; Bullard et al., 2010). Previous studies have shown that gene expression levels do not support the expected population structure compared to transcriptome variation (Wang et al., 2018), which is consistent with the present study. However, to understand the impact of genetic variation in gene expression levels and its effect on trait adaptation, it is essential to characterize expression level variation at the population scale (Fraser et al., 2011). In this study, transcript abundance was determined at the individual level using 101 wild *E. sibiricus* accessions and PSEGs were identified. In the SC, QH, XZ, and NM geo-groups, the number of PSEGs exceeded the number of selective genes identified by transcriptomic SNPs, and more genes related to local climate adaptation were discovered, such as ion transport (caused by the severe saline-alkaline soils of SC, QH, and NM) and UV-B radiation (caused by the high altitude of XZ). As the expression levels of many genes controlling quantitative traits are often influenced by regulatory genes (i.e., TFs), this study used WGCNA analysis to explore some transcription factor family members, such as NAC and zinc finger protein, that could potentially regulate PSEGs. Identification of transcriptome SNPs and PSEGs associated with adaptation can shed light on the selective pressures faced by species in their natural environment and their effects on genotype and phenotype. The integration of genotype and regulatory network (especially functional network) is a prominent and inevitable trend in current research on adaptive evolution.

## Materials and methods

### Materials and transcriptome sequencing

A total of 101 accessions covering the main distribution regions of *E. sibiricus* were included in the present study, divided into seven geographical groups, namely MGL (Mongolia), RUSS (Russia), KAZA (Kazakhstan), Canada (Canada), XJ (Xinjiang, China), NM (Inner Mongolia, China), and QTP (Qinghai, China; Sichuan, China; and Xizang, China) (**Supplementary Table S1**). Accessions from MGL, RUSS, KAZA and Canada were collected from the National Plant Germplasm System (NPGS). The remaining accessions were collected by the research team according to the following rules: Spikelets were harvested from each individual plant, ensuring a minimum sampling distance of 50 meters between plants. Seeds of all accessions were planted in sand and grown in the greenhouse to minimize the influence of the environment on the expression of the different accessions. At the three-leaf stage, leaves from each accession were collected within one hour and immediately preserved in liquid nitrogen for subsequent RNA extraction. High quality RNA was purified and fragmented after assessment of purity, quantity, and RNA integrity. First-strand cDNA was then synthesized, followed by the addition of A-tailing and adaptor. The amplified products were then purified and paired‐end sequenced to a read length of 150 bp on the Illumina platform. Finally, approximately 6 Gb of raw data was obtained for each accession, with each accession having three biological replicates.

### The assembly and annotation of the full-length transcriptome, and SNP calling

The full-length *E. sibiricus* transcriptome bam file was obtained from Yu et al. (2023) and converted to a fasta file using the bam2fasta tool (part of the bambamv1.4tool-kit) (Page et al., 2014). This was used for assembly using flye v2.9 with the following parameters: ‘flye –pacbio-raw input.fasta –out-dir out_pacbio’ (Kolmogorov et al., 2019), which uses repeat graphs for the assembly of long and error-prone reads. The quality of the assembly was accessed using QUAST (Gurevich et al., 2013). The resulting transcript sequences were aligned using the NR (Pruitt et al., 2005), COG (Tatusov et al., 2000), KOG (Koonin et al., 2004), KEGG (Kanehisa et al., 2004), and GO (Ashburner et al., 2000) databases with an *E*-value of 10-5. The transferred amino acid sequences were searched against the library of Pfam HMMs using pfam_scan.pl (Finn et al., 2014). Coding sequences (CDS) and transcription factors were then identified using the online software TransDecoder v3.0.0 (https://github.com/TransDecoder/TransDecoder) and ITAK v1.6 (Zheng et al., 2016), respectively.

Quality control was performed on the reads of the RNA sequencing data using the fastQC v0.12.0 software with the command ‘fastqc *.fastq.gz’ (https://www.bioinformatics.babraham.ac.uk/projects/fastqc/). Low quality sequences (Phred < 20 and length < 50 bp) and adaptor sequences were trimmed using trim_galore. The clean high-quality reads were compared to the full-length transcriptome data using RSEM v1.1.17 (Li and Dewey, 2011) to calculate gene abundance using default settings, which was further standardized to FPKM (fragment per kb per million reads).

Raw RNA sequencing data from one replicate of each accession were aligned to the assembled full-length transcriptome using the BWA-MEM algorithm of bwa v0.7.17-r1188 (Li, 2013; Tarasov et al., 2015) and converted to bam files using samtools v1.18 with the command ‘samtools view -b’ (Li et al., 2009), followed by the use of sambamba v0.5.9 (Li et al., 2009) to sort the resulting bam files and create index files using ‘sambamba index’. Finally, variant detection was performed using the HaplotypeCaller and GenotypeGVCFs with gatk v4.0 (McKenna et al., 2010). SNPs near indels were filtered, and the resulting vcf file was further filtered based on specific criteria: minor allele frequency (MAF) < 0.05, maximum missing data < 0.8, minimum depth of sequencing (minDP) < 2, maximum depth of sequencing (maxDP) < 1000, minimum Phred-scale quality score (minQ) < 30, minimum genotype quality score (minGQ) < 10. Finally, only biallelic loci are retained.

### Genetic diversity and population structure analysis

SNPs obtained by mapping to the full-length transcriptome (transcriptome SNPs) were used to calculate MAF, observed heterozygosity (Ho), and expected heterozygosity (He) values for each geo-group using PLINK v1.90b4.6 software (Purcell et al., 2007). In addition, transcriptome SNPs were used to perform principal component analysis (PCA) using PLINK v1.90b4.6 (Purcell et al., 2007) with visualization using the pca_plink_plot R package. Expression levels were subjected to PCA analysis using the prcomp function and then visualized using the scatterplot3d R package (Mächler and Ligges, 2003). The vcf file was sorted using tassel v5.0 software (Bradbury et al., 2007) and then converted to Phylip format using run_pipeline.pl. This was then used to construct the maximum likelihood (ML) tree using FastTree v2.1 (Price et al., 2009). The hierarchical clustering tree was constructed based on the Euclidean distance using the ggraph R package (Si et al., 2020).

Using the vcf file containing all transcriptome variations, nucleotide diversity (Pi) values of each geo-group and pairwise genetic differentiation index (Fst) were calculated using vcftools v0.1.16 (Danecek et al., 2011) with a window size of 1000 bp and a step size of 100 bp. Regions with the top 5% Fst values were identified as candidate selected regions and used to extract genes under selection. Gene flow between geo-groups was analyzed using Treemix v1.13 software (Pickrell and Pritchard, 2012) and the optimal m value was accessed using the optM R package (Fitak, 2021). Finally, the plotting_funcs.R implemented in Treemix was used for visualization.

### Population divergence in gene expression

The rsgcc R package (Ma and Wang, 2012) was used to identify PSEG with a threshold of 1. GSVA, a method for estimating variation in gene set enrichment through samples of an expression dataset, was used to determine the significantly different Gene Ontology (GO) terms between each geo-group and other accessions using the GSVA R package (Hänzelmann et al., 2013). Furthermore, the functional annotation of common specifically expressed genes in each geo-group was analyzed using the simplifyEnrichment R package (Gu and Hübschmann, 2023).

### Homologous sequences isolation based on short read sequencing

SISRS (SNP Identification from Short Read Sequences) (Schwartz et al., 2015) allows the extraction of phylogenetically relevant sites from genome sequencing data, even for low coverage sequencing (genome skimming), and is not constrained by the availability of reference genome information. The genome skimming data for the test 101 *E. sibiricus* accessions were obtained from Xiong et al. (2023), with approximately 8 Gb of raw reads (fastq format) for each accession. Given that the genome size of *E. sibiricus* is approximately 6.86 Gb based on survey sequencing (Xiong et al., 2021), the data set of approximately 800 Gb can achieve a minimum coverage of approximately 10X and was found to be sufficient for assembly of a composite genome using Velvet v1.2.10 (Zerbino and Birney, 2008). The raw data for each accession were then mapped back to the composite genome using Bowtie 2 (Langmead and Salzberg, 2012) to extract conserved and variant regions. Alignment file containing the variant information were used to perform the PCA analysis and construct the ML trees using the adegenet (Jombart, 2008) and ggtree (Yu et al., 2017) R packages, respectively.

### WGCNA analysis and network construction

WGCNA is a systems biology approach to describe the correlation patterns between genes (Langfelder and Horvath, 2008). We performed WGCNA using the WGCNA R package (Langfelder and Horvath, 2008) based on the gene expression matrix of all 101 accessions. The genes with the FPKM values greater than 0.1 in more than 95% of the samples were used for analysis (Li et al., 2020). The weighted score threshold was set to 0.8 (Wan et al., 2018; Liang et al., 2020), and then the regulatory network between transcription factor (TFs) genes and downstream genes was constructed and visualized using the ggraph R package (Si et al., 2020). The node-link graph of PSEGs was connected to show the unique regulatory network of each geo-group.

## Data Availability

The data presented in the study are deposited in the China National GeneBank DataBase repository (https://db.cngb.org/), accession number CNP0005048.
