# Supplementary material for: Extensive transcriptome data providing great efficacy in genetic research and adaptive gene discovery: a case study of Elymus sibiricus L. (Poaceae, Triticeae)
Source: Front Plant Sci. 2024 Sep 19;15:1457980. doi: 10.3389/fpls.2024.1457980 (PMC11447521; doi:10.3389/fpls.2024.1457980)
Supplement: Supplementary file 1 [file DataSheet1.zip › Additional file legend.docx]

Table S1 The origin of the tested *Elymus sibiricus* accessions. KAZA: Kazakhstan; MGL: Mongolia; RUSS: Russian; NM: Inner Mongolia, China; QH: Qinghai, China; SC: Sichuan, China; XJ: Xinjiang, China; XZ: Tibet, China.

Fig S1 The mean m (migration edges) ± standard deviation (SD) values of m values from 0 to 10 (upper chart) and the Δm chart of m values from 1 to 9 (lower chart).

Fig S2 The pairwise Fist values among the nine *E. sibiricus* geo-groups.

Fig S3 The heatmaps of the specifically-expressed-genes of each geo-group versus others.

Fig S4 Hierarchical clustering tree (dendrogram) of all genes.
