# Supplementary figures and images for: Extensive transcriptome data providing great efficacy in genetic research and adaptive gene discovery: a case study of Elymus sibiricus L. (Poaceae, Triticeae)
Source: Front Plant Sci. 2024 Sep 19;15:1457980. doi: 10.3389/fpls.2024.1457980 (PMC11447521; doi:10.3389/fpls.2024.1457980)

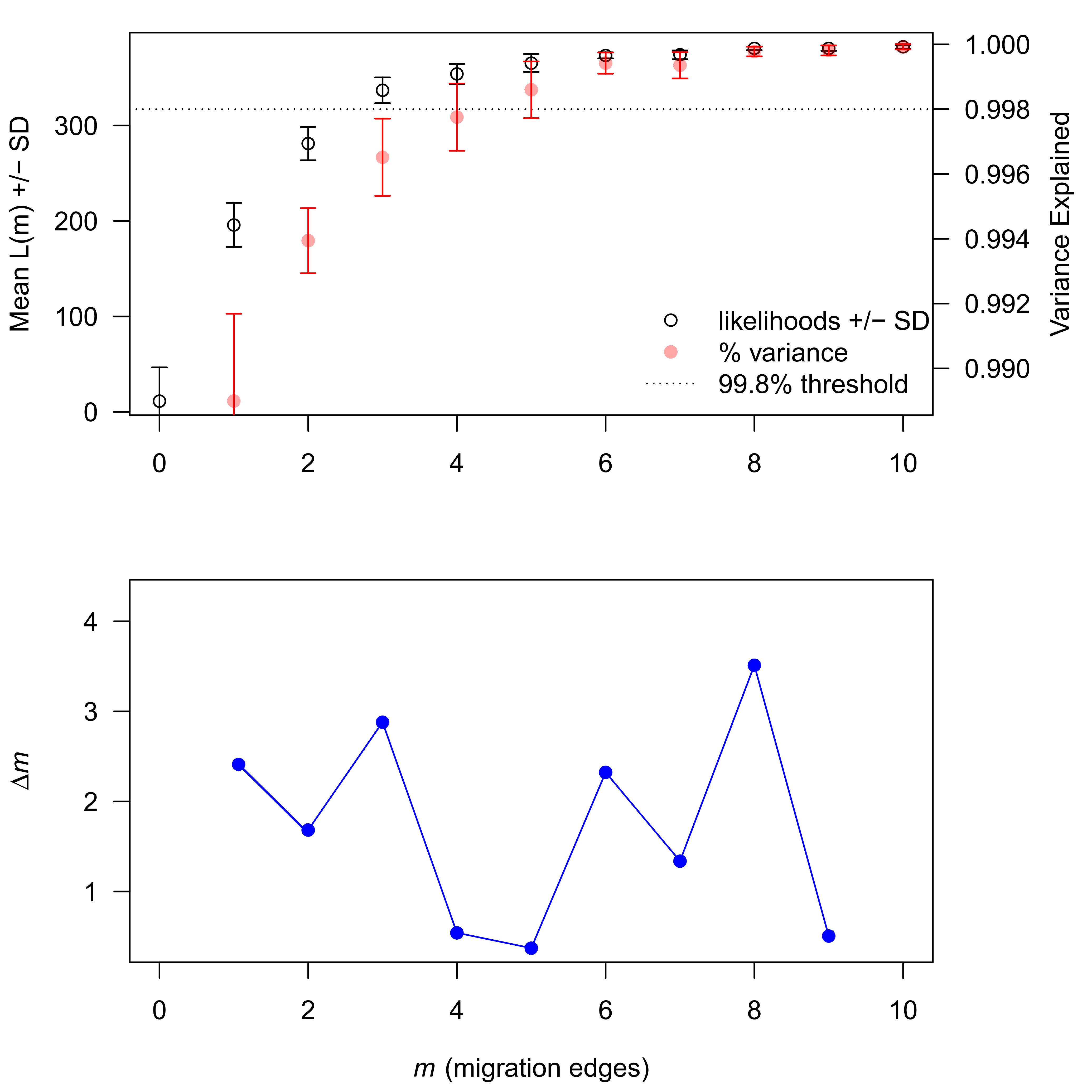

Supplement: Supplementary file 1 [file DataSheet1.zip › Fig S1.tif]

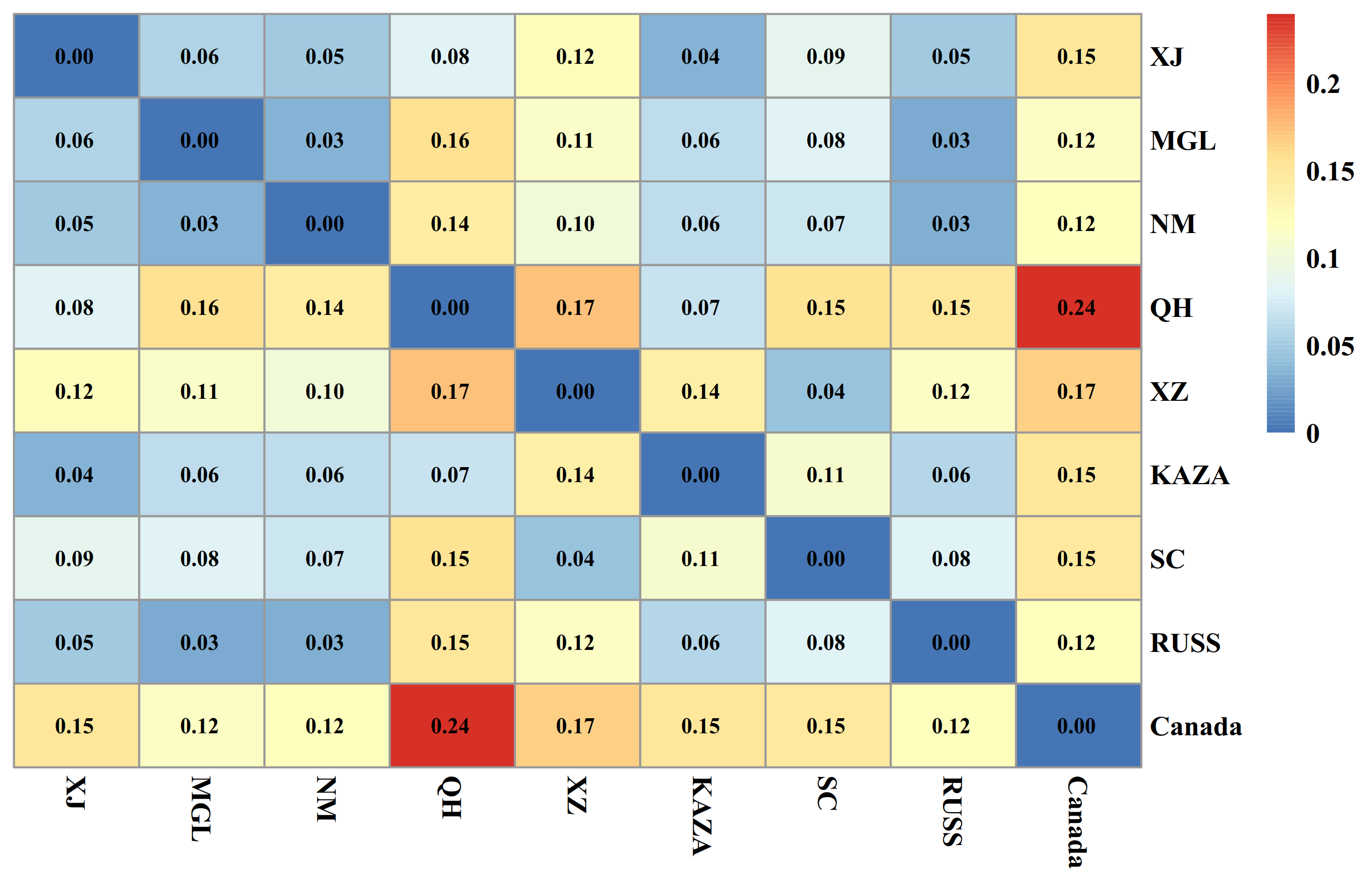

Supplement: Supplementary file 1 [file DataSheet1.zip › Fig S2.png]

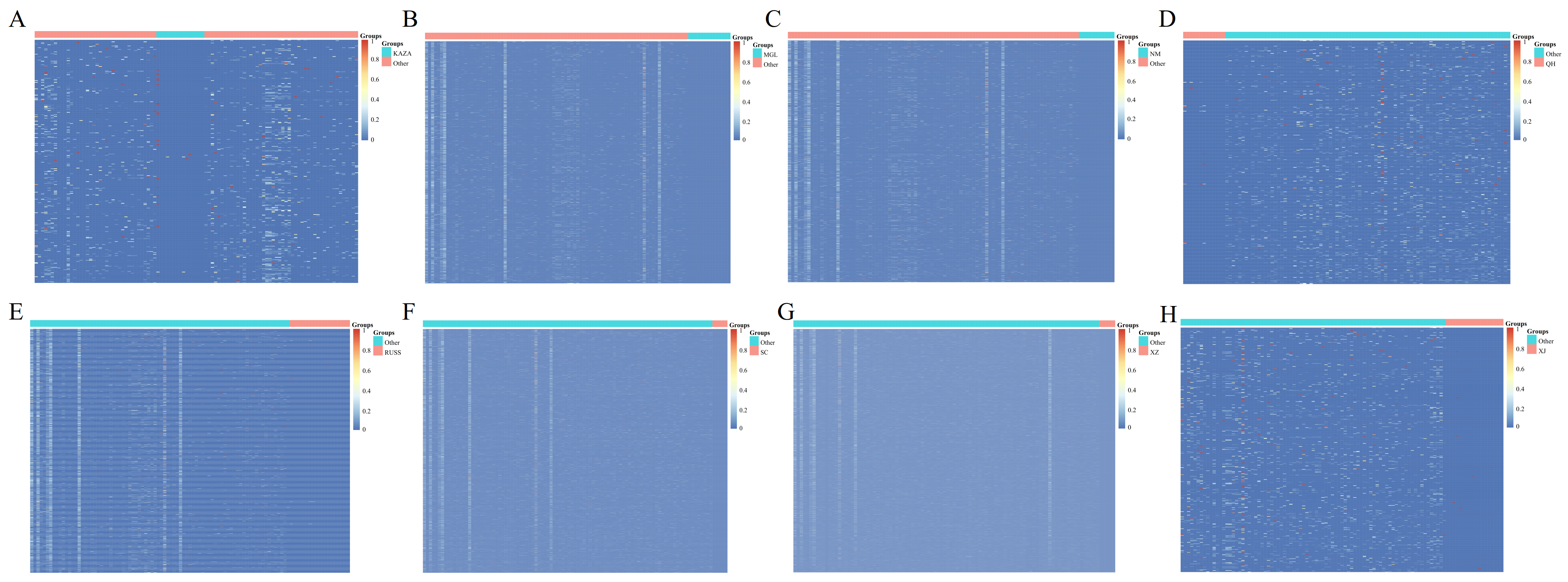

Supplement: Supplementary file 1 [file DataSheet1.zip › Fig S3.tif]

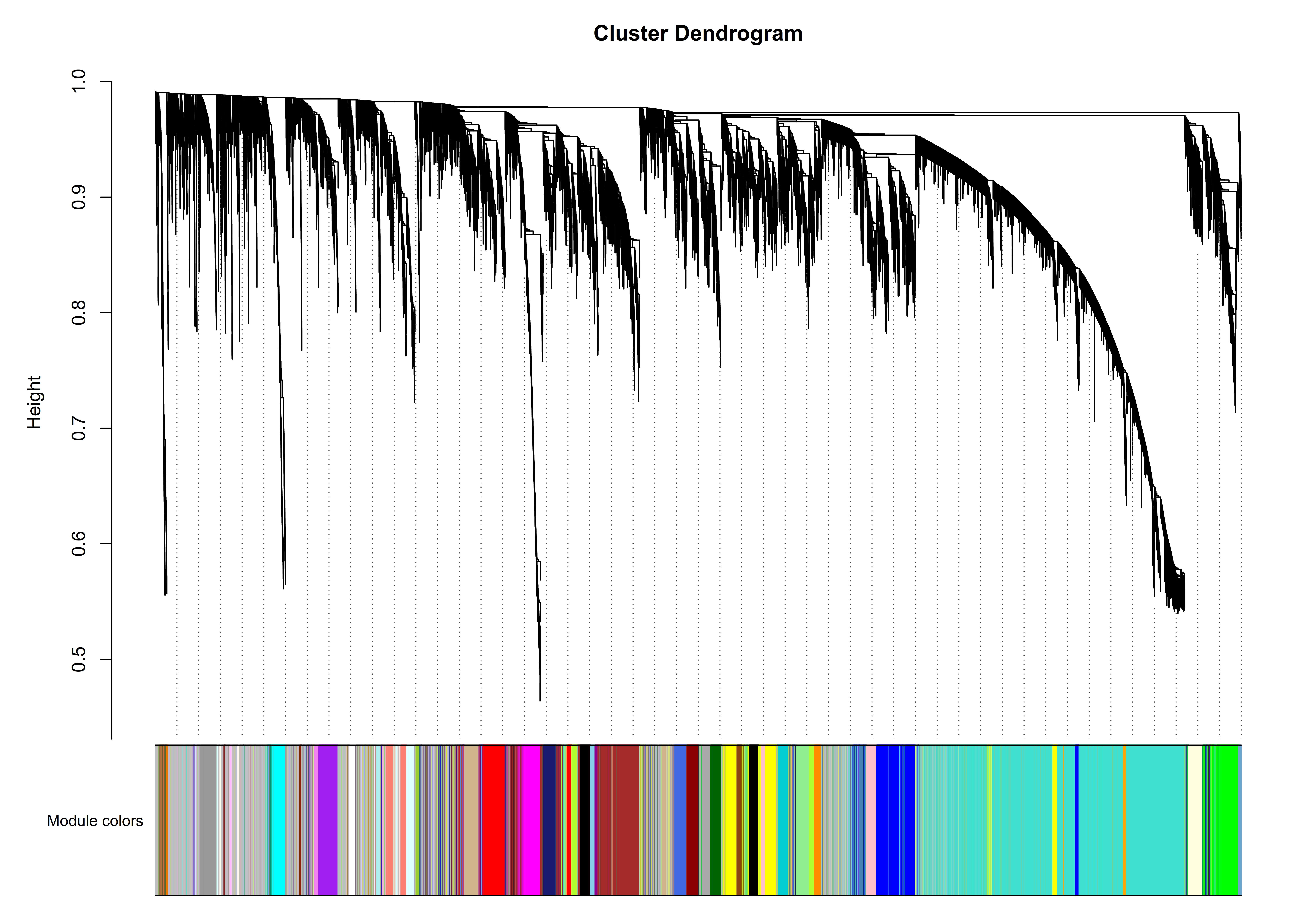

Supplement: Supplementary file 1 [file DataSheet1.zip › Fig S4.png]
